# Proteomic analysis of the response of *Funnelifor mismosseae*/*Medicago sativa* to atrazine stress

**DOI:** 10.1186/s12870-018-1492-1

**Published:** 2018-11-21

**Authors:** Xin Sui, Qi Wu, Wei Chang, Xiaoxu Fan, Fuqiang Song

**Affiliations:** 1 Heilongjiang Provincial Key Laboratory of Ecologial Restoration and Resource Utilization for Cold Region, School of Life Sciences, Heilongjiang University, XueFu Road No.74, Nangang district, Harbin City, 150080 People’s Republic of China; 2 Engineering Research Center of Agricultural microbiology Technology, Ministry of Education, Heilongjiang University, XueFu Road No.74, Nangang district, Harbin City, 150080 People’s Republic of China

**Keywords:** Arbuscular mycorrhiza, *Medicago sativa*, Atrazine, MDA, Protective enzyme system, Proteome

## Abstract

**Background:**

Arbuscular mycorrhizal (AM) fungi form symbiotic associations with host plants can protect host plants against diverse biotic and abiotic stresses, and promote biodegradation of various contaminants. However, the molecular mechanisms of how the arbuscular mycorrhizal fungi and host plant association on atrazine stress were still poorly understood. To better characterize how arbuscular mycorrhizal fungi and host plant interactions increase atrazine stress, we performed physiological and proteomic analysis of *Funneliformis mosseae* (mycorrhizal fungi) and *Medicago sativa* (alfalfa) association under atrazine stress.

**Results:**

The results showed that in the Arbuscular mycorrhizal, protective enzymes were up regulated and the malondialdehyde content increased relative to those of non-mycorrhizal *M.sativa*. We also examined the atrazine degradation rates within the nutrient solution, and a 44.43% reduction was observed with the mycorrhizal *M.sativa*, with 30.83% of the reduction attributed to *F. mosseae*. The accumulation content in root and stem of mycorrhizal *M.sativa* were obviously increased 11.89% and 16.33% than those of non- mycorrhizal *M.sativa.* The activity of PPO, POD, CAT and SOD in mycorrhizal *M.sativa* were obviously higher than non mycorrhizal *M.sativa* under atrazine stess. We identified differential root proteins using isobaric tags for relative and absolute quantization coupled with liquid chromatography–mass spectrometry, with 533 proteins identified (276 unregulated and 257 downregulated). The differentially expressed proteins were further examined using GO, BLAST comparisons, and a literature inquiry and were classified into the categories of atrazine degradation (37.1%); atrazine stress response (28.6%); plant immune responses (14.3%); translation, synthesis, and processing (10%); and signal transduction and biological processes (10%). Furthermore, we identified glycosyl transferase, glutathione S-transferase, laccase, cytochrome P450 monooxygenase, peroxidase, and other proteins closely related to the degradation process.

**Conclusions:**

Mycorrhizal *Medicago* showed improved atrazine degradation within the culturing medium and increased atrazine enrichment in the roots and stems. Additionally, AMF increased the plant root response to atrazine, with relevant enzymes up regulated and toxic effects alleviated. Overall, the findings of this study show that AMF played an important role in easing atrazine stress in plants and contributed to atrazine remediation and further contributed to the understanding of the molecular mechanism associated with atrazine stresses and potential mycorrhizal contributions in *M.sativa*.

## Background

Atrazine is an herbicide widely used in agriculture and forestry to control most broadleaf and some gramineous weeds. Atrazine applications span decades [1], despite causing serious harm to the ecosystem and with some studies indicating that the herbicide acts as an endocrine disruptor in humans and animals [2]. In China, atrazine pollution accidents have subsequently rendered large areas of farmland unusable because of toxicity and have resulted in billions of dollars of economic losses [3]. Therefore, an approach that reduces or eliminates the toxic effects of atrazine is of the utmost importance.

In previous studies that examined methods of atrazine degradation, approaches include chemical methods, such as photolysis [4] and hydro carbonylation [5]; physical methods, such as ion-exchange [6] and adsorption on activated carbon fibres [7]; and biological methods, including bacterial [8], fungal [9],and plant [10] remediation. Of these methods, the utilization of fungal–plant interactions is the most environmentally friendly. Arbuscular mycorrhizal fungi (AMF) possess several characteristics that make them attractive for use in the atrazine degradation process. First, AMF are well adapted to the environment and can withstand drought [11], soil salinization [12], heavy metals [13], and other adverse conditions [14]. Additionally, AMF can be used as a biological fertilizer in soil pollutant remediation because the physical and chemical properties of the soil improve to maintain fertility [15]. AMF also serve as a plant “organ” and expand nutrient absorption via abundant external hyphae [16]. Last, AMF can increase plant defence capabilities and mitigate damage in cases of root [17] or soil-borne [18] diseases.

At present, various studies have used mycorrhizal biotechnology in the remediation of atrazine-contaminated soil and obtained positive results [19, 20]. Previously, our lab found that the addition of *Glomus mosseae* (mycorrhizal fungi) to atrazine-contaminated soil (concentration of 50 mg/kg) achieved a 91.6% degradation rate, with 22.6% of that the mycorrhizal contribution [21]. In a similar study, transcriptome sequencing identified 172 significantly up-regulated genes closely related to atrazine degradation and discovered that extracellular enzymes are the key to atrazine degradation [22]. Additionally, late in the atrazine degradation process, with a mycorrhizal symbiont, metabolites were varied [23]. However, most studies focus on transcriptomic and metabolomic approaches, with few studies examining proteomic alterations associated with atrazine stress. When examining the mechanisms associated with plant stress resistance, many studies rely on proteomic approaches. Recently, many proteomic technologies, such as two-dimensional electrophoresis (2-DE), difference gel electrophoresis (2D-DIGE), protein arrays, label-free quantification, and isobaric tags for relative and absolute transcriptome (iTRAQ), have been utilized to identify differential expression associated with various components of animals, microorganisms, plants, and human cells [24].

In recent years, with the deepening of research on soil organic pollution remediation, the use of plant-microbial joint restoration has become an important development direction of soil bioremediation technology. Although there are many studies on AMF improving plant stress resistance, but there are still few studies on how AMF and host respond to stress, hence it is necessary to strengthen the theoretical study of mycorrhizal fungi. In this study, we utilized iTRAQ to identify differential expression in the roots of mycorrhizal *Medicago sativa* (alfalfa) relative to non-mycorrhizal *M. sativa* during atrazine stress. The aim of this study was to further examine plant responses to atrazine stress at the proteomic level and to better characterize the mechanisms associated with atrazine tolerance during a plant–fungus symbiosis. The findings presented herein provide a theoretical basis for pesticide remediation in agricultural ecosystems, aid in environmental protection, and promote the sustainable development of eco-agriculture.

## Results

### Mycorrhizal colonization rate in *M.sativa*

**Fig. 1:**
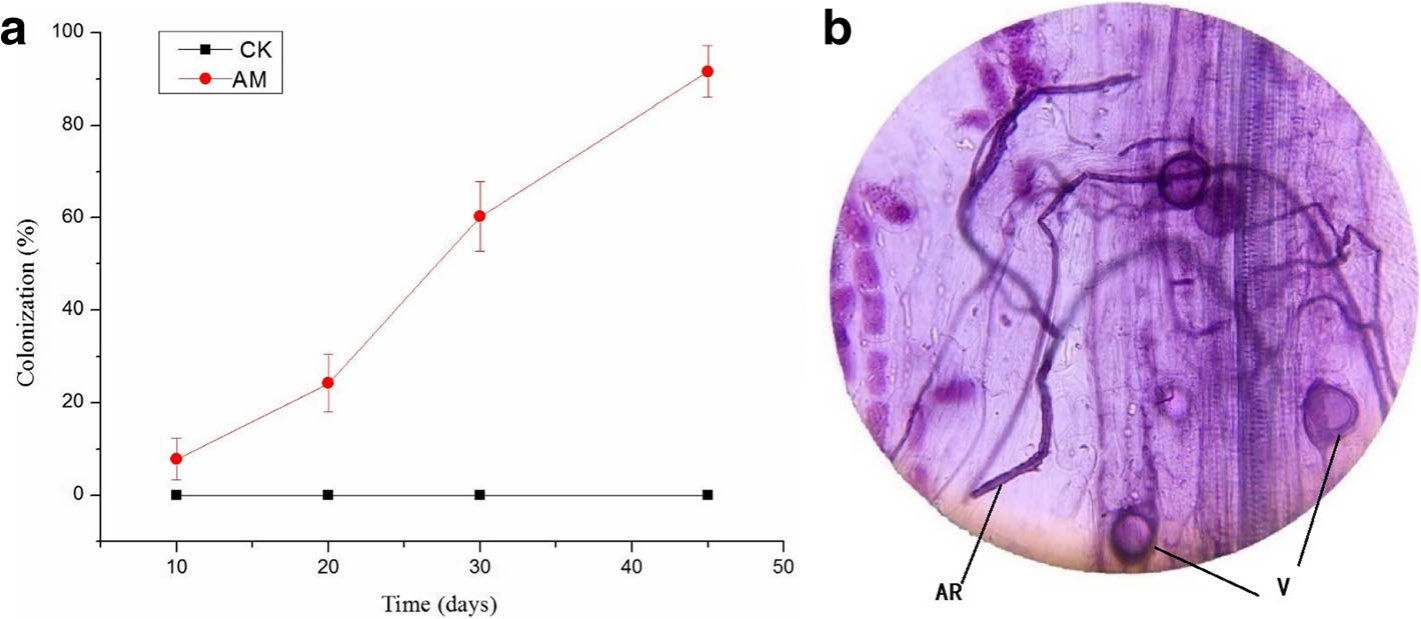
AMF colonization rate changes overtime and representative image of AMF colonization. Note: (**a**) represents AMF colonization rate changes. (**b**) represents photomicrograph of structural colonization of AMF in the root of *Glomus mosseae*. CK, non-mycorrhizal without atrazine stress (0 mg/L); AM, mycorrhizal without atrazine stress (0 mg/L); Error bars represent the standard error of mean of three replicates (*n* = 100). AR: Arbuscule; V: Vesicles

After 10 days of *G.mosseae* and *M.sativa* co-culture, only a few hyphae were detected in some roots and no vesicles appeared, thereby indicating a low infection rate. As plant growth increased, the fungal reproductive capacity also gradually increased, thereby increasing the infection rate (Fig. 1a). After 45 days, the inoculated roots were filled with hyphae and vesicles (Fig. 1b), with a mycorrhizal infection rate of 91.63%. In the CK group, no hyphae were detected, which indicated that no indigenous AMF were in the culture medium.

### Atrazine content in the nutrient solution and *M.sativa* tissue

**Fig. 2:**
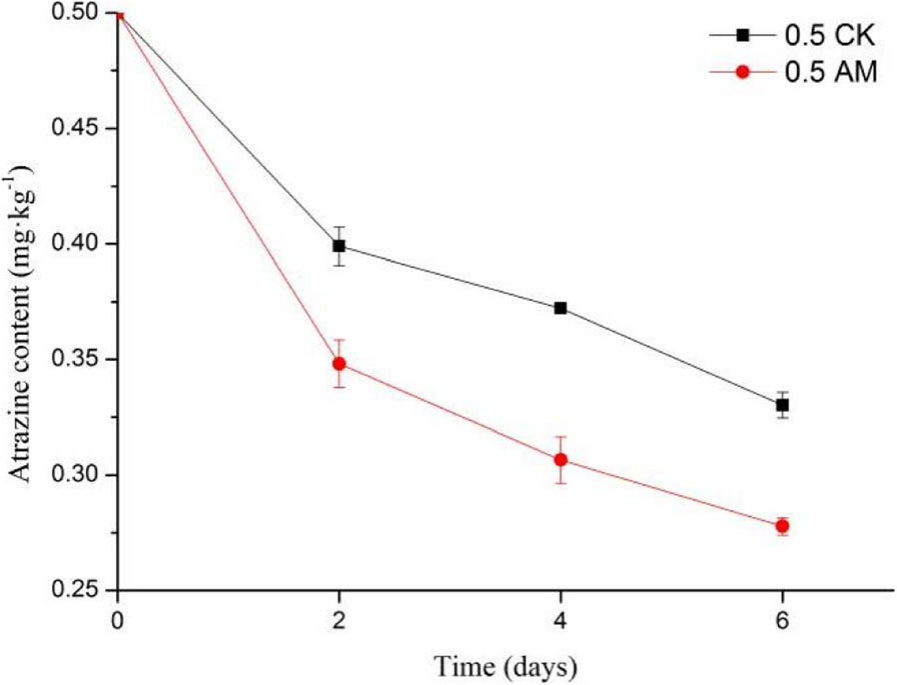
Atrazine content in the nutrient solution over time in the CK and AM groups. Note: 0.5 CK, non-mycorrhizal with atrazine stress (0.5 mg/L); 0.5 AM, mycorrhizal with atrazine stress (0.5 mg/L); Error bars represent the standard error of the means for three replicates (*n* = 3).

We measured atrazine content in the nutrient solution at 2, 4, and 6 days post-atrazine treatment in the AM and CK groups. The atrazine concentrations gradually decreased from the initial concentration of 0.5 to 0.28 mg/L in the AM group and to 0.33 mg/L in the CK group. The atrazine removal rates were 44.43% in AM and 33.96% in CK (Fig. 2), with the removal rate significantly higher in the AM than in the CK group (*p* < 0.05).

**Fig. 3:**
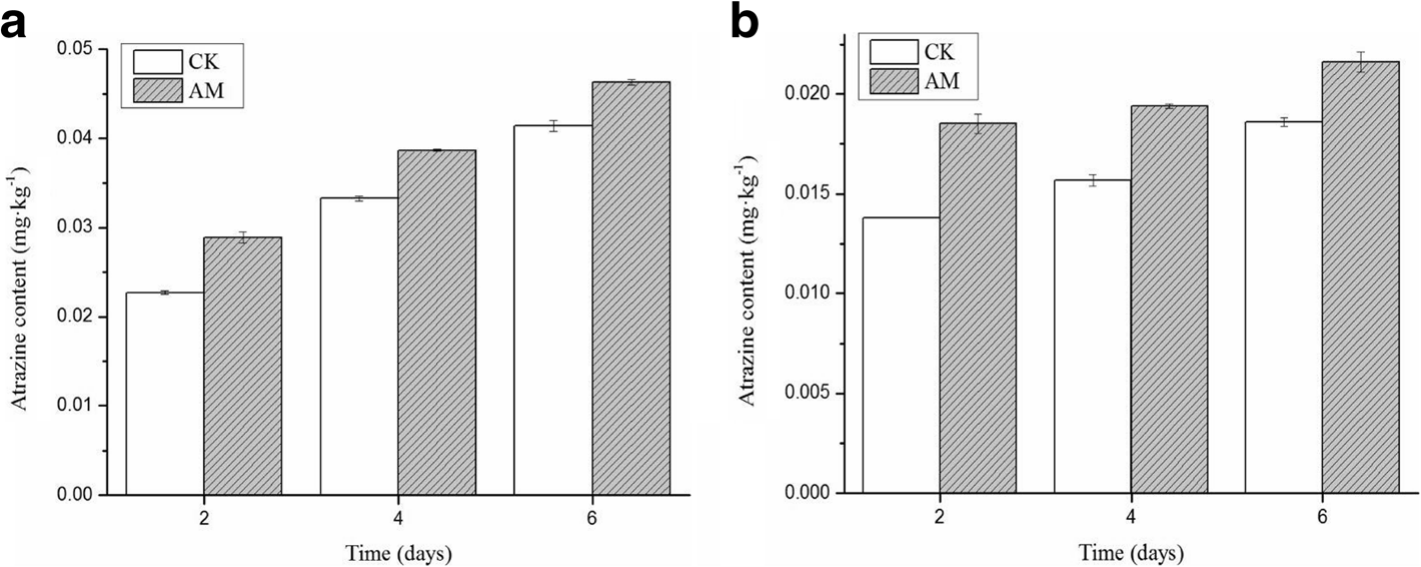
Atrazine accumulation in *M.sativa* tissues. Note: (**a**) roots and (**b**) stems. CK, non-mycorrhizal with atrazine stress (0.5 mg/L); AM, mycorrhizal with atrazine stress (0.5 mg/L); Columns represent the means for three replicates (n = 3). Error bars show the standard error.

Additionally, we examined atrazine accumulation in roots and stems (Fig. 3), and levels increased significantly on days 4 and 6 compared with those on day 2 (*p* < 0.05). The roots absorbed more atrazine than the stems in both groups, but the AM group had higher levels in the roots (11.89%) and stems (16.33%) overall by day 6 than those in the CK group.

To show the changes in atrazine accumulation or metabolism directly, we determined bioaccumulation coefficient (BCF) and transfer coefficient (TF) values (Table 1). In the AM group, the BCF values of the roots and stems were 0.17 and 0.08, respectively, and in the CK group, they were 0.13 and 0.06, respectively; which were not significantly different. However, these findings suggested that the reduction of atrazine in the culture medium was predominately due to plant or natural degradation rather than absorption by the plant. When examining the TF values, the AM group had a higher value than that of the CK group, which showed that the AM group was better able to shift the pesticide from the underground region to the upper region. These results show that AMF can improve the ability of plants to absorb, transport, and metabolize atrazine.

**Table 1:** The BCF and TF indexes of atrazine in *M.sativa* tissues (6 days post-treatment)

Treatments	BCF		TF
	Stem	Root	
CK group	0.06^b^	0.13^b^	0.45^b^
AM group	0.08^a^	0.17^a^	0.47^a^

*p* < 0.05 and *n* = 3

### Examination of malondialdehyde (MDA) and praline (pro) content following atrazine stress

**Fig. 4:**
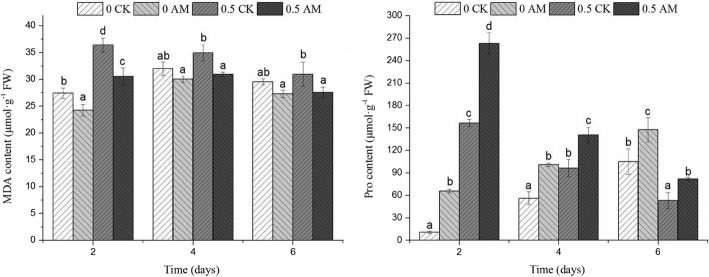
Examination of MDA and Pro content in *M.sativa* roots during atrazine stress. Note: 0 CK, non-mycorrhizal without atrazine stress (0 mg/L); 0 AM, mycorrhizal without atrazine stress (0 mg/L); 0.5 CK, non-mycorrhizal with atrazine stress (0.5 mg/L); 0.5 AM, mycorrhizal with atrazine stress (0.5 mg/L); Columns represent the means for three replicates (n = 3). Error bars show the standard error. Columns with different letters indicate significant differences between treatments at *P* < 0.05

During atrazine stress, the MDA and Pro contents in the roots of both groups showed a downward trend with time (Fig. 4). By day 6 post-atrazine treatment, the MDA content of the AM group was significantly lower than that of the CK group (*p* < 0.05; 11.03%), whereas the Pro content was significantly higher than that of the CK group (*p* < 0.05; 53.96%). Before reaching day 6, the MDA content in the AM group (7.52%) was lower than that in the CK group, and the Pro content increased by 40.32% compared with that of the CK group.

### Examination of root protective enzyme activities during atrazine stress

**Fig. 5:**
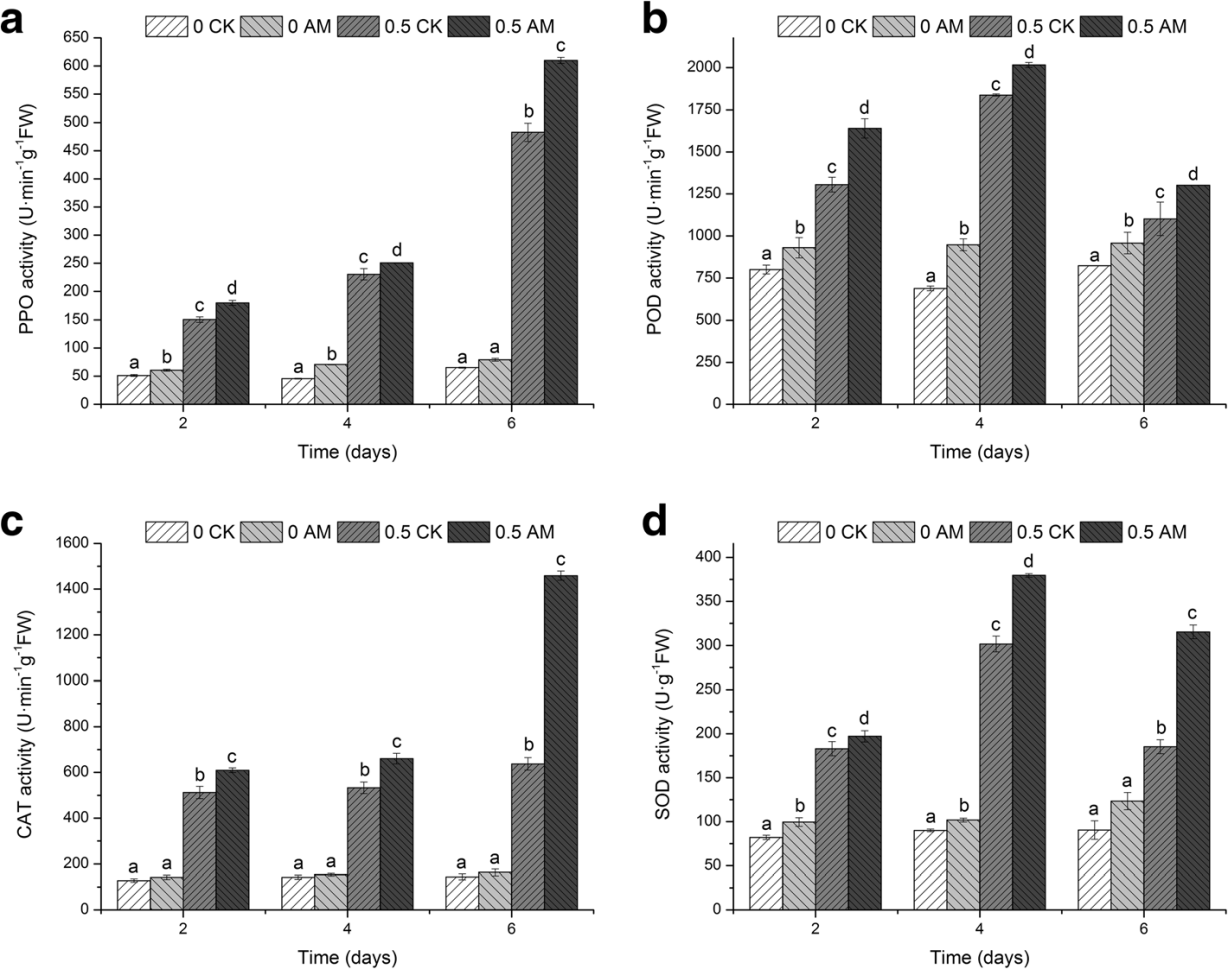
Changes in (**a**) PPO, (**b**) POD, (**c**) CAT, and (**d**) SOD activities in different days. Note: 0 CK, non-mycorrhizal without atrazine stress; 0 AM, mycorrhizal without atrazine stress; 0.5 CK, non-mycorrhizal with atrazine stress (0.5 mg/L); 0.5 AM, mycorrhizal with atrazine stress (0.5 mg/L); Columns represent the means for three replicates (n = 3). Error bars show the standard error. Columns with different letters indicate significant differences between treatments at *P* < 0.05

We examined four stress-related enzymes: polyphenoloxidase (PPO), peroxidase (POD), catalase (CAT) and superoxide dismutase (SOD) (Fig. 5). PPO is a type of copper binding enzyme that is widely found in plants, animals, and fungi and participates in biological oxidation. When an organism is experiencing stress, PPO is in an active state. Following atrazine stress, root PPO levels showed an upward trend (Fig. 5a), with the AM group showing significantly higher levels than those of the CK group by day 6 (*p* < 0.05; 26.31). When examining the AM and CK groups not exposed to atrazine stress, PPO activity remained consistent throughout, and no significant differences were noted between the groups.

The enzyme POD was also examined, which is closely associated with plant photosynthesis and respiration intensity. POD, via an H_2_O_2_ electron acceptor, removes phenol and amine substances that are harmful and reduces H_2_O_2_, phenol, and amine toxicity. In *M.sativa* roots under atrazine stress, POD activity levels increased first and then decreased (Fig. 5b); with the activity in the AM group significantly higher than that in the CK group throughout (*p* < 0.05) and 37.51% higher by day 6. In the groups not exposed to atrazine stress, POD showed a slight upward trend, with POD levels in the AM group significantly higher than those in the CK group (*p* < 0.05).

CAT plays an important role in biological defences and is key to the maintenance of low H_2_O_2_ levels. CAT catalyses the decomposition of H_2_O_2_ into water and molecular oxygen, with iron porphyry as the auxiliary base, thereby reducing toxicity. During atrazine stress, the root CAT activity in the AM group showed a significant upward trend relative to that in the CK group (*p* < 0.05; Fig. 5c), with levels 41.2% higher by day 6. In the groups not under atrazine stress, CAT activity did not change significantly (*p* < 0.05).

SOD is a metal-containing enzyme that removes active oxygen free radicals, thereby preventing functional, compositional, and structural oxidative damage. In roots under atrazine stress, SOD activity levels first increased and then decreased (Fig. 5d). At day 4, activity levels peaked and then dipped on day 6, with activity levels significantly higher in the AM group than those in the CK group (*p* < 0.05; 70.29%). In the groups with no atrazine stress, SOD activity was significantly higher in the AM group than that in the CK group for all time points except for day 6 (*p* > 0.05). These findings suggest that during atrazine stress, many reactive oxygen species are produced; thus, PPO, POD, CAT, and SOD activities increase to offer a protective effect. The higher levels of enzyme activities observed in the AM than in the CK group suggested that those plants more effectively alleviated atrazine stress.

### The identification of differential proteins during atrazine stress

**Fig. 6:**
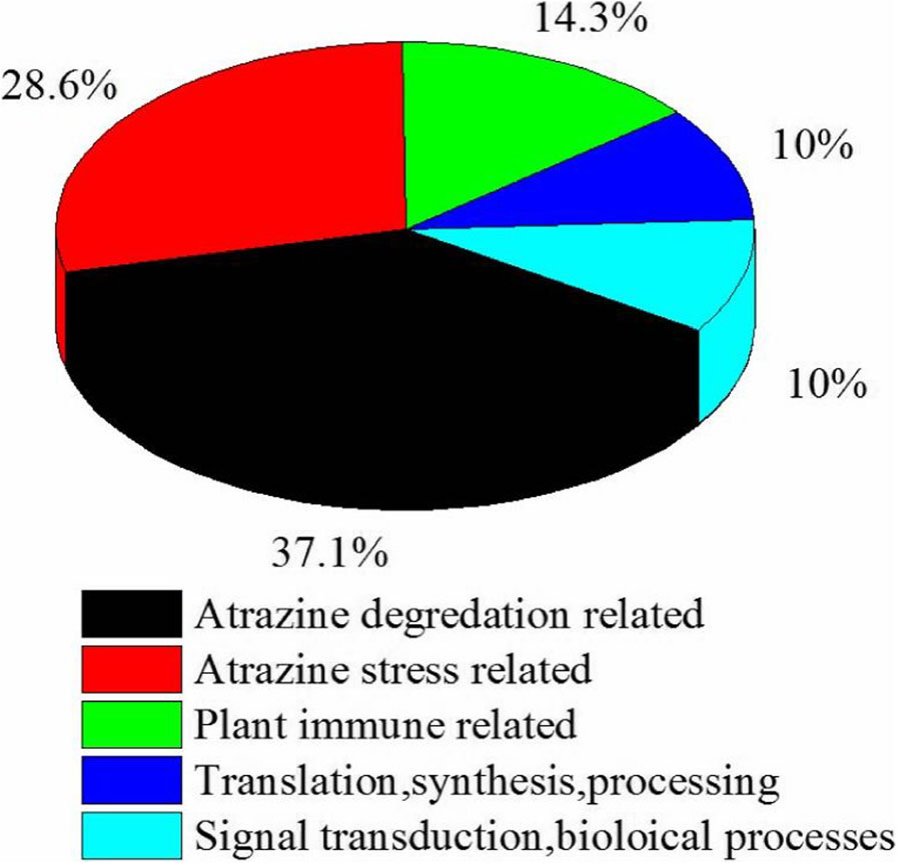
Functional classification of differential AM proteins

We identified differential proteins using iTRAQ coupled with liquid chromatography–mass spectrometry (LC-MS), with 533 proteins identified in total. Significantly up-regulated proteins had to meet the condition of AM/CK > 1.2, whereas significantly down-regulated proteins had to satisfy AM/CK < 0.83.A total of 533 proteins were differential proteins, with 276 up regulated and 257 down regulated. Following GO and BLAST analyses (Fig. 6), these proteins were classified into five categories, including atrazine biodegrading (37.1%); proteins related to atrazine stress response (28.6%); proteins that participate in plant immune responses (14.3%); protein translation, synthesis, and process-related proteins (10%); and proteins associated with signal transmission and biological processes (10%; see Table 2).

**Table 2:** Functional classifications of the differential proteins

Accession	Protein Name	Organism Name	Gene Name	MW[kDa]	Calc.pI	Fold Change AM/CK	t-test *p*-value
Atrazine degradation related proteins							
A0A072TVX3	Cytochrome P450 family protein	*Medicago truncatula*	MTR_8g100135	46.013	8.221	2.075	0.021
A0A072TSI8	Cytochrome P450 family protein	Medicago truncatula	MTR_0155s0010	16.067	9.232	0.732	0.017
Q1WCN7	Cytochrome P450 monooxygenaseCYP83G2	Medicago truncatula	CYP83G2	57.618	7.738	1.524	0.013
G7KEE9	Cytochrome P450 family 71 protein	Medicago truncatula	CYP83G1	57.712	8.280	1.437	0.029
A0A072TFX3	Glycosyl transferase	Medicago truncatula	MTR_0184s0030	52.474	5.630	1.341	0.009
A0A072UA34	Glycosyl transferase	Medicago truncatula	MTR_6g038200	51.461	5.656	1.276	0.043
A0A072UQY5	Glycosyl transferase	Medicago truncatula	MTR_4g117890	54.533	6.668	0.723	0.004
G7LES1	Glycosyl transferase	Medicago truncatula	MTR_8g077590	52.569	5.833	0.788	0.010
A0A072UGV0	Glutathione S-transferase, amino-terminal domain protein	Medicago truncatula	MTR_4g019780	25.264	5.275	1.339	0.013
A0A072U4C4	Glutathione S-transferase	Medicago truncatula	MTR_8g087425	25.900	5.935	1.419	0.035
G7JPE9	Glutathione S-transferase, amino-terminal domain protein	Medicago truncatula	MTR_4g059730	25.366	6.024	1.223	0.035
A0A072V9X8	Glutathione S-transferase, amino-terminal domain protein	Medicago truncatula	MTR_2g070200	25.768	8.163	0.789	0.013
A0A072UHQ4	Laccase	Medicago truncatula	MTR_4g019225	63.545	6.976	1.812	0.014
G7ILB5	Laccase	Medicago truncatula	MTR_2g008330	65.655	8.690	1.951	0.017
G7IBW1	Peroxidase	Medicago truncatula	MTR_1g098320	15.921	9.173	2.711	0.002
G7IBT2	Peroxidase	Medicagotruncatula	MTR_1g086320	36.283	8.880	2.896	0.003
A0A072U8W1	Peroxidase	Medicago truncatula	MTR_6g048160	9.618	4.716	1.248	0.014
A0A072UAE2	Peroxidase	Medicago truncatula	MTR_6g043240	38.215	8.265	1.209	0.018
A4UN77	Peroxidase	Medicago truncatula	PRX2	38.283	7.518	1.344	0.024
G7KFM2	Peroxidase	Medicago truncatula	MTR_5g074970	35.772	9.291	1.226	0.028
A0A072V2Y0	Peroxidase	Medicago truncatula	MTR_4g125940	29.259	5.300	1.209	0.037
Q43790	Peroxidase	*Medicago sativa*	prx1B	38.168	6.874	1.263	0.037
G7JIS0	Peroxidase	Medicago truncatula	MTR_4g083710	34.802	8.221	1.329	0.041
A0A072VDU0	Peroxidase	Medicago truncatula	MTR_1g009750	35.901	8.749	0.729	0.027
A0A072VLG5	Peroxidase	Medicago truncatula	MTR_1g077000	36.249	6.551	0.771	0.031
I3S041	Peroxidase	Medicago truncatula		34.816	7.137	0.825	0.033
Atrazine stress related proteins							
G7KMG8	Kunitz_type trypsin inhibitor/Alpha-fucosidase	Medicago truncatula	MTR_6g059410	22.217	5.440	1.625	0.017
G7KMU3	Kunitz_type trypsin inhibitor/Alpha-fucosidase	Medicago truncatula	MTR_6g059680	23.778	5.364	0.641	0.004
A0A072TS76	Kunitz_type trypsin inhibitor	Medicago truncatula	MTR_0211s0080	21.407	5.186	3.921	0.004
A0A072UV79	2OG-Fe(II) oxygenase family oxido reductase	Medicago truncatula	MTR_3g045230	39.822	5.364	1.661	0.006
A0A072U5A4	Chitinase/Hevein/PR-4/Wheat win2	Medicago truncatula	MTR_7g115220	21.656	6.138	1.820	0.015
B7FM99	Chitinase/Hevein/PR-4/Wheat win2	Medicago truncatula	MTR_6g082480	10.003	9.115	1.258	0.018
B8Y647	Class IV chitinase	Medicago sativa	Chi	30.466	4.856	1.362	0.024
P94084	Class I chitinase	Medicago sativa		34.900	7.562	1.491	0.024
G7ID31	Chitinase	Medicago truncatula	MTR_1g099310	31.418	5.148	1.510	0.032
G7JYI2	Heat shock cognate 70 kDa protein	Medicago truncatula	MTR_5g066800	70.343	8.617	1.342	0.046
G7ZZY8	Heat shock cognate 70 kDa protein	Medicago truncatula	MTR_4g079590	68.755	6.049	0.761	0.010
A2Q3S0	Heat shock protein Hsp70	Medicago truncatula	MtrDRAFT_AC155888g17v2	65.470	6.566	0.781	0.040
G7LII1	Legume lectin beta domain protein	Medicago truncatula	MTR_8g068040	28.106	6.379	1.866	0.024
G7IXH3	Legume lectin beta domain protein	Medicago truncatula	MTR_3g047140	30.793	5.135	1.204	0.049
A0A072TKU6	Defensin-like protein	Medicago truncatula	MTR_8g010320	7.751	8.060	1.641	0.027
G7J9K5	1-aminocyclopropane-1-carboxylate oxidase	Medicago truncatula	MTR_3g083370	35.620	5.389	1.746	0.030
Q19PX3	1-aminocyclopropane-1-carboxylate oxidase	Medicago sativa	ACO	36.070	5.186	1.672	0.005
G7KJU7	1-aminocyclopropane-1-carboxylate oxidase	Medicago truncatula	MTR_6g092620	34.351	5.960	1.445	0.023
G7KVR7	Polygalacturonase inhibitor	Medicago truncatula	MTR_7g023740	41.710	8.089	1.439	0.005
A0A072V5F9	Polygalacturonase inhibitor protein	Medicago truncatula	MTR_3g437820	24.216	8.470	1.742	0.008
Plant immune response associated proteins							
B7FI20	Syntaxin of plants 122 protein	Medicago truncatula	MTR_1g056550	34.826	8.514	1.204	0.004
A0A072VKC4	Syntaxin of plants protein	Medicago truncatula	MTR_1g066460	29.428	5.300	0.818	0.013
G7IKJ7	Syntaxin of plants 122 protein	Medicago truncatula	MTR_2g088700	34.482	6.668	0.819	0.017
P93333	Class-10 pathogenesis-related protein 1	Medicago truncatula	PR10–1	16.647	4.729	1.247	0.000
G7JLL1	Pathogenesis-related protein betVI family protein	Medicago truncatula	MTR_4g120760	17.800	4.640	1.250	0.009
A0A072U0G0	Pathogenesis-related protein betVI family protein	Medicago truncatula	MTR_8g045555	17.609	6.037	0.529	0.005
A0A072TYD0	Caffeic acid O-methyltransferase	Medicago truncatula	MTR_8g024160	36.298	5.389	2.025	0.004
G7 L368	Caffeic acid O-methyltransferase	Medicago truncatula	MTR_7g012070	41.776	5.732	1.323	0.012
G7 L359	Caffeic acid O-methyltransferase	Medicago truncatula	MTR_7g011990	40.692	5.440	1.222	0.018
I3SBC6	CAP, cysteine-rich secretory protein, antigen 5	Medicago truncatula	MTR_2g435490	20.192	8.221	1.726	0.010
Proteins involved in translation, synthesis and processing							
G7LDS2	30S ribosomal protein S20	Medicago truncatula	MTR_8g061350	19.108	10.580	1.346	0.024
A0A072VKA9	50S ribosomal protein L11P	Medicago truncatula	MTR_1g060610	16.183	9.994	0.629	0.006
A0A072URU1	60S ribosomal protein L24–2	Medicago truncatula	MTR_4g104930	7.537	10.287	0.640	0.011
B7FMT1	30S ribosomal protein S17	Medicagotruncatula	MTR_2g101770	16.642	9.906	0.740	0.026
G7ILF2	60S ribosomal protein L26–1	Medicago truncatula	MTR_2g012450	16.653	11.151	0.706	0.029
G7K2J3	60S ribosomal proteinL26–1	Medicago truncatula	MTR_5g015570	16.674	11.019	0.805	0.040
G7IDU4	Protein disulfide isomerase-like protein	Medicago truncatula	MTR_1g087520	40.402	5.491	1.222	0.015
Proteins associated with signal transduction and biological processes							
P93330	Nodulin-13	Medicago truncatula	N13	18.169	4.678	1.792	0.012
G7KGN2	Leghemoglobin Lb120–1	Medicago truncatula	MTR_5g041610	16.174	6.329	1.643	0.003
G7IRQ5	Serine/Threonine kinase family protein	Medicago truncatula	MTR_2g094090	45.759	5.389	1.227	0.014
A0A072TPB2	AT hook motif DNA-binding family protein	Medicago truncatula	MTR_8g036160	37.118	8.851	0.440	0.004
A0A072VXL7	AT hook motif DNA-binding family protein	Medicago truncatula	MTR_1g076710	35.930	8.163	0.803	0.004
G7KG44	AT hook motif DNA-binding family protein	Medicago truncatula	MTR_5g011520	34.158	7.005	0.606	0.012
A0A072TPA5	AT hook motif DNA-binding family protein	Medicago truncatula	MTR_8g036060	32.093	7.606	0.720	0.020

## Discussion

### Effects of atrazine on the physiological indexes of *M. sativa*

Previous studies suggest that MDA, Pro, and antioxidant activity within the root system during adverse stress are an indication of the ability to manage pollutant stress at different levels [25], with Pro having a buffer effect during stress [26]. In this study, plants rapidly synthesized large amounts of Pro at the start of atrazine stress, with levels then decreasing over time. At first, these findings appear in contrast to previous findings [27]; however, the prolonged atrazine stress might have exceeded tolerance, which led to metabolic and osmoregulatory disorders that resulted in a loss of proline.

Levels of MDA and PPO are used as indicators of the degree of cell membrane damage and degree of stress [28] and in this study, indicated that cell membrane damage occurred during atrazine stress. The MDA content decreased with increased time of stress, indicating that the atrazine affects were better tolerated with time [29], whereas SOD, PPO, POD, and CAT continuously increased. Previous studies show that after stress induction, plant antioxidant enzyme activity first increases and then decreases [30]. However, the findings in this study differed, which suggested that *M.sativa* resistance mechanisms during atrazine stress did not depend entirely on antioxidant enzyme activity. Additionally, the root Pro levels were consistently higher in the AM group than in the CK group, whereas the MDA content was always lower than that of the CK group. These findings suggested that AMF played an important role in alleviating atrazine stress, which is consistent with previous studies that show AMF can increase stress resistance [31, 32].

### Differential protein expression associated with atrazine stress

When plants enter symbiosis with AMF, the process is continuous and dynamic and includes the stages of pre-symbiosis, early symbiosis, and symbiotic maturity [33], with the development of special mycorrhizal structures and physiological characteristics [34]. In this study, we identified differentially expressed proteins associated with atrazine stress using iTRAQ coupled with LC-MS. The identification of these proteins contributes to a better understanding of the resistance mechanism associated with atrazine stress.

### Proteins associated with atrazine biodegradation

Of the differential proteins associated with mycorrhizal *Medicago*, 26 were associated with atrazine biodegradation and included cytochrome P450 monooxygenase, glycosyltransferase, glutathioneS-transferase (GST), laccase, and peroxidase. Currently, various studies show that the P450 gene is directly involved in the process of pesticide degradation [35, 36] and significantly improves tolerance to substances such as isopropyl methylamine, atrazine, and ethylamine [37]. Additionally, P450 is essential in the biosynthesis of plant photochemical compounds and plays a crucial role in resisting pathogens [38].

Glycosyltransferase also plays an important role in regulating exogenous pollutants [39] by improving the target pollutant hydrophilicityvia UDP-glucose, which is regarded as an aglycosylation donor. This alteration then activates membrane transport and influences solubility to ultimately turn the toxic target pollutant into a low-toxicornon-toxic substance [40, 41]. During the early stage of atrazine stress, previous studies find that glycosyl transferase transcriptional levels increase [21]. In this study, protein levels varied; thus, further exploration into the role of glycosyltransferase in atrazine degradation is required.

The GST gene family encodes enzymes that degrade harmful exogenous chemicals [28] and serve as antioxidants [42], and these enzymes are found in wheat, sorghum, alfalfa, and other crops [43]. In this study, three GST proteins were up regulated and one was down regulated. These results may be associated with the *M. sativa* activating mechanisms that are associated with reactive oxygen species in response to atrazine stress and result in increased antioxidant levels and their associated mechanisms.

During pesticide stress, biodegrading enzymes, such aslaccase, peroxidase, catalase, lipase, and glutaredoxin [44, 45], are secreted. In this study, two laccases and nine peroxidases were up regulated in the AM group relative to these enzymes in the CK group. Laccase predominantly participates in lignin synthesis but also has a close association with plant disease resistance [46, 47]. In one study, the GaLAC1 gene from cotton was transfected into *Arabidopsis thaliana* and increased eugenic acid and mustard acid tolerance [48]. In a study examining transgenic tobacco that contained a tomato TPX2 peroxidase gene, the transgenic variety showed increased ability to retain water with improved germination rate and salt resistance [49]. Additionally, in ginkgo exposed to heavy metal pollution, the ginkgo peroxidase gene (GbPOD1) was implicated in the defence and removal of the heavy metals [50]. Furthermore, our previous study also showed a similar expressional trend for these two types of enzymes [22]. Although these enzymes appeared to contribute to the synergistic degradation of atrazine and protect the plant at the physiological and protein levels, further examination is required to determine their exact mechanisms.

### Proteins associated with atrazine stress responses

Kunitz-type trypsin inhibitors can inhibit protease hydrolysis and protect other defensive proteins from degradation, in addition to also regulating endogenous protease activity and preventing disease [51]. One study found that when cotton cultivars were transfected with soybean Kunitz-type trypsin inhibitors, they were more resistant to pesticides [52]. Therefore, Kunitz-type trypsin inhibitors have the potential to increase pesticide resistance when utilized in genetic engineering.

Plant growth can be modulated via methylation levels during external stress. In this study, the 2OG-Fe (II) oxygenase family of oxidoreductases increased in the AM group, which is involved in the process of converting 5-mctransmethylation into 5-hmC and 5-fc. To some extent, the genes within this family are present across many protein networks [53] and co-coordinate plant stress responses.

Chitinase catalyses the synthesis of chitin and dextran linear polysaccharide chains in plants and also plays an important role in the process of fungal infection [54]. In the present study, chitinase was up regulated in the AM group relative to that in the CK group both with and without atrazine stress, which indicated that the mycorrhizal association might contribute to an increase in stress resistance.

Heat shock proteins aid in damage protection and can even repair damaged proteins [55]. One study showed that heat shock proteins play an important role in resisting exogenous organic compounds, such as anthracene and pyrene [56]. Furthermore, one study showed that two wheat heat shock proteins offered protection in the presence of organic toxicants [57]. In the body, these proteins participate in protein folding and assembly and strengthen the ability to respond to external stress. In this study, heat shock expression increased in mycorrhizal *Medicago*, thereby improving atrazine tolerance.

Lectin, which can recognize mannose, chitin, and t-antigens, is an important glycoprotein that increases during mycorrhizal establishment and promotes physiological plant functions [58]. In white kidney beans, lectin may provide an inhibitory effect against melon blight and grape grey mildew [59]. Similar to lectin, defensin-like proteins also serve in a protective capacity, and a sweet pepper defensin element transformed into *E.coli* conferred antibacterial activity [60]. In this study, a defens in-like protein and lectin were up regulated in the AM group, which suggested that the mycorrhizal symbionts had increased tolerance to atrazine stress.

Ethylene, which is important in fruit maturation, is formed when 1-aminocyclopropane-1-carboxylate oxidase associates with catalysed 1-aminocyclopropane-1-carboxylicacid (ACC) [61]. In one study, weeds expressing the ACC gene were resistant to the hormone herbicide chloroquinoline [62]. Another protein that also functions similarly to ACC is the polygalacturonase inhibitor protein, which is as pecificheat-stablegly co-protein. Additionally, one study found a correlation between responses to endurance to mechanical injury and low temperature stress [63]. In this study, the polygalacturonase inhibitor protein and 1-aminocycloproclopropane-1-carboxylateoxidase were up regulated in the AM group and presumably contributed to atrazine tolerance.

### Plant immune response-related proteins

When a plant is exposed to viruses or organic toxicants, the immune response is stimulated. The syntaxin protein (SYP), which is a member of the SNARE (soluble N-ethyl-male antibody-sensitive fusion protein attachment protein) protein family, functions in vesicle-mediated transport in plant cells. The SYP gene is associated with plant abiotic stresses, cytoplasmic division, gravity response, lipi drafting, and plant disease resistance [64]. In one study, the loss of the AtSYP121 gene due to bacterial infection resulted in an increase in fungal penetration and the destruction of plant viral defences [65]. Moreover, the AtSYP121 gene is implicated in the salicylicacid, jasmonicacid, and ethylene-dependent defence pathways.

Pathogenesis-related protein (PR)is induced by a variety of exogenous stimulating factors (pathogens and organic pollutants, among others) and endogenous factors (plant hormones, ethylene, salicylic acid, and hydrogen peroxide, among others). The expression of this protein is closely related with plant protoplast embryogenesis, the germ formation of the callus, and the regulation of hormones (auxin IAA and cytokine in CTA) in the process of germ formation [66, 67]. The accumulation of PR protein is significantly correlated with stress intensity [68]. In this study, two PR proteins were up regulated and one was down regulated in the AM group. Mycorrhizal associations allow plants to respond quickly to stress [69], but in this study, the PR levels were only gradually reduced rather than completely reduced, possibly because of a gradual decrease in the basic metabolic capacity and productivity of the plant. Therefore, we speculate that the observed PR expression in the AM group alleviated the effects of atrazine stress on plant growth.

The biological function of lignin includes limiting the mechanical damage to plant cells and tissues and also forming a natural barrier that can hinder various pathogens. Caffeic acid O-methyltransferase (COMT) is a key catalytic enzyme in the process of plant lignin synthesis. Lignin content is modulated by various transcription factors, such as NAC, MYB, and zinc finger transcription factors [70]. When the COMT gene or protein expression is down regulated, the lignin content is also reduced, and plant resistance to exogenous substances is weakened. In this study, COMT expression was up regulated in the AMF symbionts, which suggested an increase in resistance to atrazine stress relative to that of the control group. Cysteine-rich secretory proteins (CRISPs) are found in the mammalian reproductive tract, with their expression level closely associated with the regulation of the human immune system and a variety of major human diseases. COMT also has been examined as a potential biomarker and drug therapy target [71]. Although not well characterized in plants, COMT is regulated by negative feedback and produces osmotic substances and detoxification substances and regulates closure to reduce cell membrane injury [72]. In this study, AMF symbionts showed an up-regulation of COMT, suggesting a protective function during atrazine stress.

### Proteins relating to translation, synthesis, and processing

The processes of translation, synthesis, and processing are subjected to various levels of regulation. Ribosomal proteins exist in the ribosome, and their synthesis is controlled by the activity of the GTP enzyme. The functions of ribosomal proteins are complicated and not fully elucidated, but how the ribosome forms during translation is clear. One study reported an up-regulation of ribosomal proteins when wheat under high temperature stress was treated with growth regulators (coronamic acid, COR) [73]. In this study, ribosomal proteins were up regulated in the AM group relative to that in the control, indicating increased protein synthesis. Protein disulfide isomerase-like protein (PDIL) has a disulfide bond that inhibits the formation of erroneous positions between cysteines by regulating the dox pathway in the cytoplasm during rapid peptide folding [74]. In our study, PDIL was unregulated in the AM group during atrazine stress, which indicated a suppression of erroneous positions during semi synthesis and increased adapt ability during stress.

### Proteins associated with signal transduction and biological processes

Plants associated with AMF show in creased ability to resist biological or abiotic stresses. Mycorrhizal associations involve complex recognition signals and a complex biological metabolism [75, 76]. In this study, Nodulin13 expression was unregulated, which indicated that the mycorrhizal symbionts could degrade atrazine. Leghemoglobin Lb120–1 is a pigment in plant nodules that is similar to vertebrate my glob in and plays an important role in plant nitrogen fixation, nodulation, maintenance of the intracellular environment, and ATP production [77]. The soybean plant Lb120–1 expression is not detected until rhizobia nodule formation is detected [78]. In the AM group, compared with the CK group, Lb120–1 expression was unregulated, which suggested that this group was more robust and therefore more able to tolerate atrazine stress.

Serine/threoninekinase (STK)–related proteins function in signalling and are involved in the physiological responses of most cells [79]. Previous studies show that STKs are involved in plant defence responses, with activity modulated by low temperature, soybean mosaic viral infection, or other factors [80]. In this study, STK expression was unregulated in the AM group, which indicated an atrazine tolerance. AT-hook plays an important role in transcriptional regulation by serving as acofactor modulating chromosomal structure and as a transcription factor [81]. DNA-binding proteins containing AT-hook motifs are found in humans, insects, and plants [82]. One study examining the AT-hook gene family in rice found expression during development, particularly in the rice seedling stage, indicating a role in plant growth and metabolism [83]. In this study, AT-hook expression was down regulated in the AM group, which suggested a possible alternative function in stress response, but further examination is required.

### Network interaction predictions based on differential expression

**Fig. 7:**
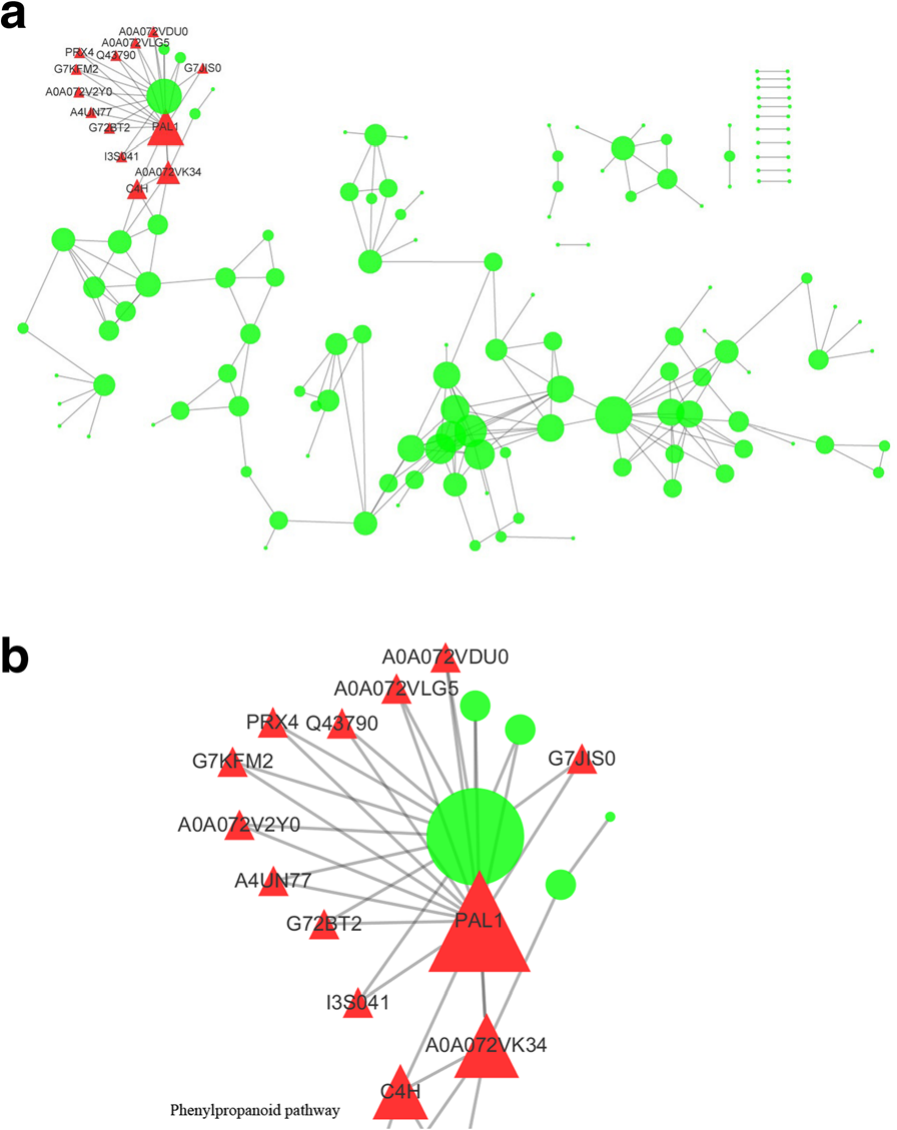
Protein-interaction network interactions for differentially expressed proteins. Note: **a** shows the integrated network for all the differentially expressed proteins; **b** shows the network interactions for the differentially expressed proteins related with phenylpropanoid pathway. Red triangle represents the differentially expressed proteins related with phenylpropanoid pathway; Green circle represents other differentially expressed proteins, respectively. The sizes represent the abundance of differentially expressed proteins

**Fig. 8:**
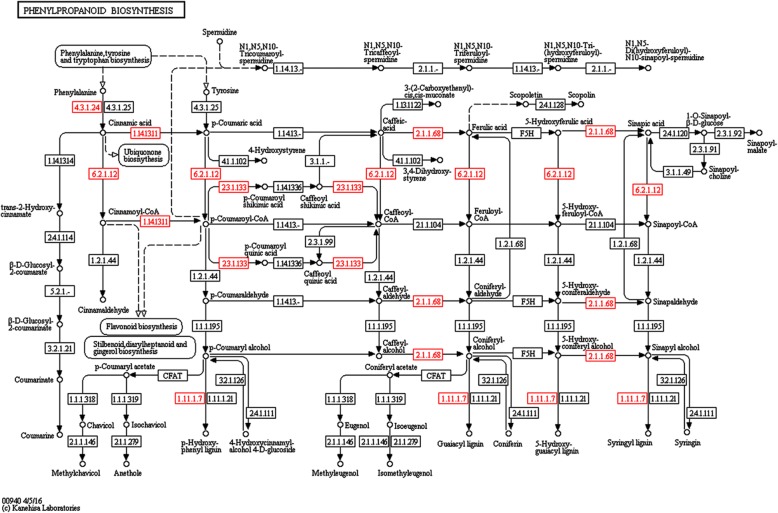
Synthetic pathway of phenylpropanoid compounds. Note: Pathway Projector provides an integrated pathway map that is based upon the KEGG Atlas. Red boxes are KEGG enzymes coded at high enrichment

We predicted target proteins using PPI analysis in CytoScape (3.2.1), with the target proteins (direct and indirect interactions) located in the IntAct database. These interactions included the direct physical proteins and the indirect proteins correlated with indirect functions. The key proteins that affected the entire metabolic system or signal transduction pathways are shown in Fig. 7. The results showed that phenylalanine ammonia-lyase protein (PAL-1) was the most correlated protein, directly or indirectly, with connections to proteins such as peroxidase, GLYMA02G40040.1, trans-cinnamate 4-monooxygenase, and GLYMA15G13510.1. Additionally, most of the target proteins were associated with PAL1 and were in the phenylpropanoid biosynthesis pathway (Fig. 8); thus, this pathway might play an important role in stress tolerance. Phenylalanine ammonia-lyase has a special sub cellular localization and can function in cellular metabolism, plant development, and other biological transmission processes. Phenylalanine ammonia-lyase interacts with various peroxidases, thereby participating in alfalfa phenylpropanoid biosynthetic processes, and modulates phenyl propane metabolites and stress responses. These findings showed that the atrazine stress response is a multi-factor process involving many protein interactions.

### Molecular patterns of AM-*M. sativa* response to atrazine stress

**Fig. 9:**
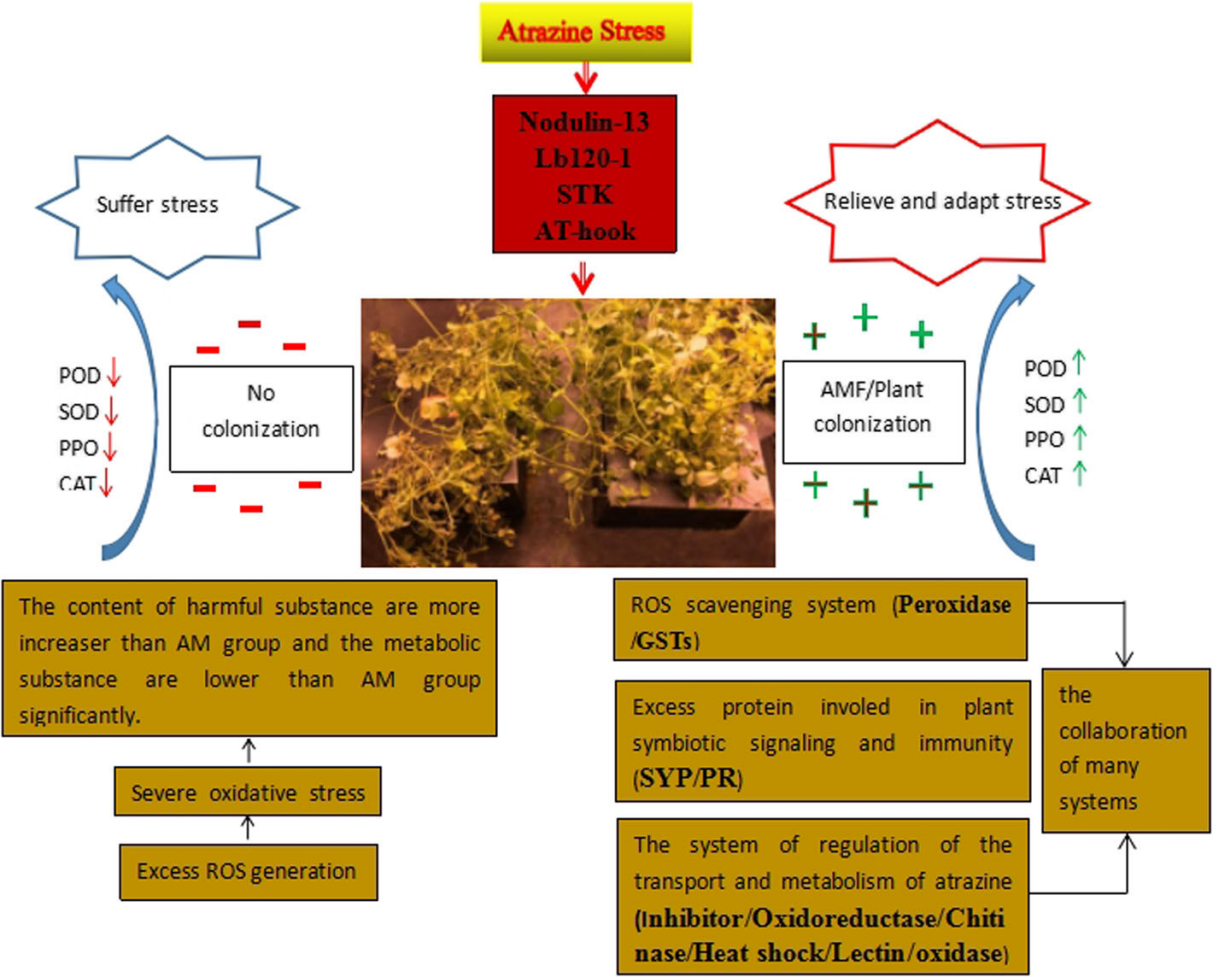
A schematic of mycorrhizal plant responses to atrazine stress. The red arrow and green arrow indicate increased and decreased proteins, respectively. POD: peroxidase; SOD: superoxide dismutase; PPO: polyphenolox; CAT: catalase

Molecular modelling of mycorrhizal and non-mycorrhizal *M. sativa* under atrazine stress was conducted (Fig. 9). The right side of the figure represents the AM group under atrazine stress, the left side depicts the CK group under atrazine stress, and the middle represents the physical properties. When atrazine stress began in the AM group, expression was altered relating to regulatory factors (Nodulin-13, Lb120–1, STK, and AT-hook), specific proteins (CRISPs, COMP, and ribosomal), degradation proteins (oxidoreductase, chase, oxidative, lectin, and oxidase), and immune proteins (SYP and PR), thereby promoting stress tolerance. Additionally, the physiological indicators showed that antioxidant enzymes increased, thereby decreasing peroxide products and improving the osmotic adjustments. The ability of AMF to induce atrazine resistance in *M. sativa* at the protein level was primarily achieved by the regulation of the antioxidant system, transport of harmful substances and metabolic system, the signal delivery system, and the immune system. The associations and timing related to these physiological processes require further examination.

## Conclusions

*Medicago sativa* growth was significantly inhibited by atrazine, with growth improved in mycorrhizal symbionts. Furthermore, mycorrhizal *M.sativa* showed improved atrazine degradation within the culturing medium and increased atrazine enrichment in the roots and stems. Additionally, AMF increased the plant response to atrazine, with relevant enzymes up regulated and toxic effects alleviated. The actions of AMF were primarily mediated by the antioxidant system, transport and metabolic systems, and signal delivery system. Overall, these findings show that AMF played an important role in easing atrazine stress in plants and contributed to atrazine remediation.

## Methods

### Pot experiment design

Atrazine (99% purity) was purchased from China Bailingwei (Shijiazhuang City, China), and the atrazine commodity drug (38% purity) was purchased from Autumn Pesticide Ltd. (Habin City, China). *Funneliformis mosseae* (mycorrhizal fungi) was maintained in sand or soil as hyphal segments or a spore mixture, with as pore content program of inoculums of approximately 30, by the Study on Ecological Restoration of Heilongjiang University. The Heilongjiang Academy of Agricultural Sciences provided the seeds for the host plant *M.sativa*.

**Fig. 10:**
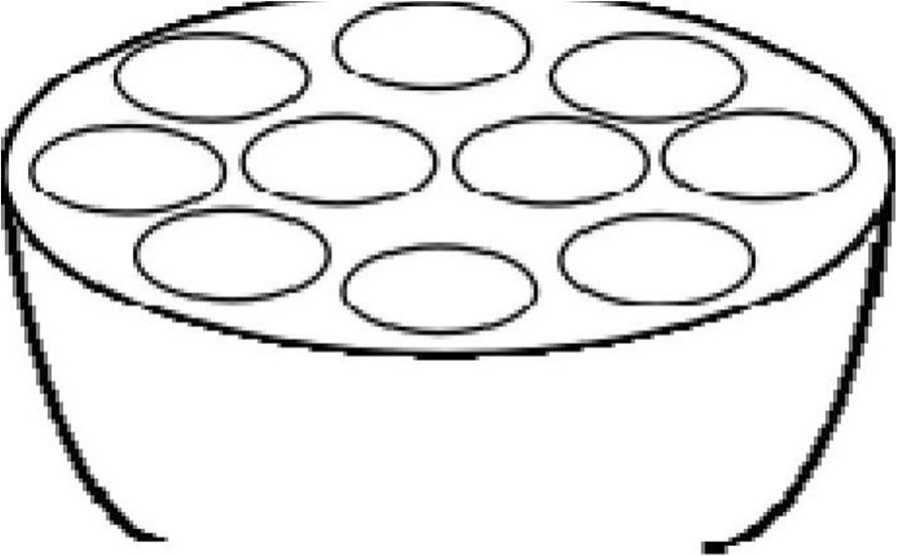
Schematic diagram of the culturing system

*Medicago sativa* seeds were rinsed in distilled water two or three times, and then we surface sterilized the seeds with 10% hydrogen peroxide (H_2_O_2_) for 10 min and laid the seeds flat on a sheet containing moist double layers of gauze for 24 h. We selected the long white seeds for potting in the culturing system (Fig. 10). The system consisted of 10 growth chambers (PVC tube; diameter = 4.5 cm, depth = 15 cm) and a fixed, blank part outside the growth chamber (PVC tube; diameter = 35 cm, height = 20 cm). Before experimentation, we sterilized the surface with 0.3% potassium permanganate. The soil was composed of a mixture of peat, vermiculite, and sand (5:3:2 mix) and was passed through a 10-mesh sieve, sterilized at 121 °C for 2 h, and treated with 5% sterilization agent. Samples were divided into two groups: an AM group that contained *F. mosseae* and a CK group that did not. To ensure that *M.sativa* roots could be separated from the culture medium, we assessed the chamber before planting. We filled the fixed chamber of the culture system with the prepared culture medium, inserted the PVC tubes of the 10 growth chambers into the stroma, and passed distilled water through the soil to confirm stability. Finally, we evenly distributed 50 germinated seeds into each growth chamber and covered them with a thin layer of soil. Displaced light at an illumination intensity of 350 μmol m^− 2^ s^− 1^ was provided daily, with a continuous illumination of 14 h. Day and night temperatures were 25 °C and 20 °C, respectively, with the relative humidity maintained at 65%.

### Sample collection and preparation

*Medicago sativa* seedlings were grown for 40 days, with plants whose roots had reached the bottom of the culture system removed from the soil and washed with distilled water. We then transferred these seedlings with a similar level of growth to a black glass tank half-filled with Hoagland nutrient solution. We transplanted 15 *M.sativa* seedling stains into each pot and allowed them to adapt to the new environment for 7 days, with the nutrient solution replaced every 2 days. Next, half of the samples were treated with Hoagland nutrient solution containing 0.5 mg/L atrazine (0.5 CK and 0.5 AM groups) based on previous findings [84], whereas the other half received only Hoagland solution (0 CK and 0 AM groups) for 6 days. The roots were collected at days 2, 4, and 6 post-atrazine stress induction for both groups.

### Determination of mycorrhizal colonization rate in *M.sativa*

To assess the rate of mycorrhizal colonization, we performed acid fuchsin staining as previously described [85]. We randomly selected samples from each group, stained 100 fragments, observed and counted hyphae, and determined the infection rate.

### Determination of malondialdehyde content in *M.sativa* roots

To assess the degree of stress, we examined malondialdehyde (MDA) using the thiobarbituric acid method. Samples were taken during the same growth period and from the same root positions (0.3 g each). We performed the specific method of determining MDA content as previously described [86], with the MDA concentration (μmol/L) based on fresh weight per gram.

### Examination of the protective enzyme system in *M.sativa* roots

To further examine the effect of atrazine stress, we examined polyphenoloxidase (PPO) activity using the catechol method and peroxidase (POD) activity using the guaiacol method [87]. Absorbance was measured at 525 nm and 470 nm, respectively, with one unit (U) of enzyme activity defined as a change of 0.01 OD per minute per gram fresh weight. Catalase (CAT) activity was examined using ultraviolet absorption, with absorbance measured at 240 nm. One unit (U) was defined as a reduction in absorbance of 0.1 perminute [88]. We examined superoxide dismutase (SOD) activity using the nitrogen blue four triazole method [88], with absorbance measured at 560 nm. One unit (U) was defined as the amount of enzyme that inhibited the photochemical reduction of nitro blue tetrazolium (NBT) by 50% [88].

### Determination of atrazine content in nutrient solution and alfalfa tissue

We extracted atrazine from the nutrient solution [89] and plants [90, 91] as previously described with some modifications. A high-performance liquid chromatography (HPLC) column (D600; Waters Company, Milford, MA, USA) was utilized with a 5 μm hypersilODS2 filter (4.6 mm × 250 mm; Yilite Analytical Instrument Co. Ltd.),with 20 μL of sample load and run at room temperature. The mobile phase was composed of methanol and water (4:1), and a flow rate of 0.8 mL/min was utilized, with a detection wavelength of 220 nm.

### Bio concentration and transfer coefficients

We calculated the bio concentration coefficient as follows: bioaccumulation coefficient = atrazine concentration in plants (roots or stems)/atrazine concentration in nutrient solution. We calculated the transfer coefficient as follows: transfer coefficient = plant stem atrazine concentration/plant root atrazine concentration.

### *M.sativa* root protein extraction

After six days of atrazine stress, significant differences in morphology were noted between the experimental groups. In the CK group, 50% of the seedling leaves appeared yellow, whereas in the AM group, leaves were a scattered yellow. Proteins were extracted from root tissues using trichloroacetic acid/acetone precipitation, and SDT cleavage was utilized as previously described [92]. Protein quantification was performed via BCA. Proteins were visualized via 12.5% sodium dodecyl sulfate polyacrylamide gel electrophoresis (constant current of 14 mA for 90 min), with 20 g of protein combined with 5 μL of loading buffer and placed in a boiling water bath for 5 min before loading. Upon completion, we stained the gel with Coomassie brilliant blue to visualize the proteins.

### Enzymatic hydrolysis,iTRAQ labelling, and fractionation of *M.sativa*root proteins

We performed protein enzymatic hydrolysis as previously described [93]. The obtained peptide segments (100 μg) from each sample were then labelled using iTRAQ reagents according to the manufacturer’s instructions (Applied Biosystems, Waltham, MA, USA).We used an AKTA purifier 100 (GE Life Sciences, Chicago, IL USA), with elution first performed with buffer A (10 ml MKH_2_PO_4_ and 25% ACN; pH 3.0) at a flow rate of 1 mL/min. Elution was performed next with buffer B (10 ml MKH_2_PO_4_, 500 m MKCl, and 25% ACN; pH 3.0). We performed this process at 214 nm, with elution fractions collected every 2 min and a total of 10 replicates obtained.

### Masss pectrometry and functional annotation of *M.sativa* root proteins

We performed HPLC using an Easy-nLC system (Thermo Fisher Scientific, Colorado Springs, CO, USA) with an auto sample rata increasing flow rate. First, the column was equilibrated using 95% solution A (0.1% formic acid aqueous solution) and then at a flow rate of 300 nL/min. We desalted the samples on an Acclaim PepMap 100 column C18 (Thermo Fisher Scientific; 100 μm × 2 cm) and separated the samples on an Easy-nLC II analytical column (Thermo Fisher Scientific) via elution gradient with solution B (0.1% formic acid acetonitrile aqueous solution). Following chromatography, we performed mass spectrometry using a Q-Exactive mass spectrometer (Thermo Fisher Scientific), a positive ion detection method. The parameters were as follow: apparent ion scanning range of 300–1800 m/z, a first-order resolution of 70,000 at 200 m/z, an AGC target of 3e6, a first-level maximum IT of 10 ms, as can range number of 1, and a dynamic exclusion of 40.0 s. We identified and quantified proteins using Mascot 2.2 and Thermo Proteome Discover version 1.4 software.

### Data processing and analysis method

The data were analysed via analysis of variance using the SPSS 20.0 statistical software package (Chicago, IL, USA). We then visualized differentially expressed proteins using Cytoscape software, with values corresponding to node size, and Origin Pro 8.5.1. Values were considered significant when *p* < 0.05.

## Abbreviations

2D-DIGE: Difference gel electrophoresis
2-DE: Two-dimensional electrophoresis
ACC: 1-aminocyclopropane-1-carboxylicacid
AMF: Arbuscular mycorrhizal fungi
CAT: Catalase
COMT: Caffeic acid O-methyltransferase
COR: Coronamic acid
GbPOD1: Ginkgo peroxidase gene
GST: GlutathioneS-transferase
iTRAQ: Isobaric tags for relative and absolute transcriptome
LC-MS: Liquid chromatography–mass spectrometry
MDA: Malondialdehyde
NBT: Tetrazolium
PAL-1: Phenylalanine ammonia-lyase protein
PDIL: Protein disulfide isomerase-like protein
POD: Peroxidase
PPO: Polyphenoloxidase
PR: Pathogenesis-related protein
SNARE: Soluble N-ethyl-male antibody-sensitive fusion protein attachment protein
SOD: Superoxide dismutase
STK: Serine/threonine kinase
SYP: Syntaxin protein
